# Alcohol brief interventions in youth and social work settings in Scotland

**DOI:** 10.1186/1940-0640-8-S1-A52

**Published:** 2013-09-04

**Authors:** Tessa Parkes, Martine Stead, Douglas Eadie, Avril Nicoll, Jennifer McKell, Linda Bauld, Sarah Wilson, Cheryl Burgess, Garth Reid, John McAteer, Ruth Jepson

**Affiliations:** 1 University of Stirling, School of Nursing Midwifery and Health, Inverness, Scotland, UK

## 

Scotland has one of the highest liver cirrhosis mortality rates in Western Europe. The Scottish government has invested in a range of policies to address this and the wider harms from alcohol, including a national programme on alcohol brief interventions (ABIs). The initial focus of this work was primary care, accident and emergency care, and antenatal care but it was expanded in 2012 to include ABIs delivered in wider settings and with populations such as social work service users and young people. This process evaluation aims to explore the feasibility and acceptability of ABIs delivered to young people and in social work settings. The study involves two phases: one that maps existing projects providing ABIs in these areas and examines barriers and facilitators to their delivery, and a second that explores case study projects in depth and develops proposals for a potential future outcome evaluation. Phase 1 of the study involved conducting 24 semi-structured interviews with 28 professionals from 12 projects providing ABIs in the wider settings of social work and young people's services between December 2012 and April 2013. Two field visit observations were also completed and documentation/data gathered from all projects, including numbers of clients and ABIs delivered where possible. A framework approach was used for coding and analysis of data. In addition to a detailed thematic analysis, 10 project case summaries were produced to retain the specificity of information about the diversity and similarities across the cases studied. Results from Phase 1 of the study will be presented and implications for policy and practice will be discussed.

